# Detecting Abnormal Neuronal Activity in a Chronic Migraine Model by Egr1-EGFP Transgenic Mice

**DOI:** 10.3389/fnins.2021.705938

**Published:** 2021-08-12

**Authors:** Fei Wang, Weiqing Jiang, Li Gao, Chen Liu, Mingzhu Deng, Xiao Ren, Chenlu Zhu, Ji-Song Guan, Yonggang Wang

**Affiliations:** ^1^Department of Neurology, Renji Hospital, School of Medicine, Shanghai Jiao Tong University, Shanghai, China; ^2^Department of Neurology, Beijing Tiantan Hospital, Capital Medical University, Beijing, China; ^3^Department of Neurology, The First Affiliated Hospital of Zhengzhou University, Zhengzhou, China; ^4^Department of Neurology, The Second Hospital of Lanzhou University, Lanzhou, China; ^5^School of Life Sciences and Technology, ShanghaiTech University, Shanghai, China; ^6^CAS Center for Excellence in Brain Science and Intelligence Technology, Chinese Academy of Sciences, Shanghai, China; ^7^Headache Center, China National Clinical Research Center for Neurological Diseases, Beijing, China

**Keywords:** chronic migraine, early growth response gene 1, brain regions, neuronal activity, photophobia, psychiatric comorbidity

## Abstract

Chronic migraine (CM) is a highly disabling neurological disorder characterized by recurrent headache accompanied by a variety of sensory and/or emotional symptoms. However, the mechanisms of migraine onset and its chronicity have not been elucidated. The present study was designed to search for brain regions and neurons that were abnormally activated by CM and might be related to its pathogenesis and different concomitant symptoms. CM models were established here by repeated intraperitoneal injection of nitroglycerin (NTG) every other day for 9 days to early growth response gene 1 (Egr1)-enhanced green fluorescent protein (EGFP) transgenic mice, which allowed monitoring of neuronal activities in the whole brain. CM-related behaviors were recorded through head grooming test and light aversion assay. Elevation of Egr1 expression signals was detected in trigeminal nucleus caudalis (TNC), primary somatosensory cortex (SSp), lateral amygdala nucleus (LA), primary visual area (VISp), and temporal association areas (TEa) 2 h after the last injection of NTG by immunofluorescence and digital slice scanning technology. Meanwhile, no change of Egr1 expression was found in auditory areas (AUD), CA1, ectorhinal area (ECT), piriform (PIR), and anterior cingulate area (ACC). Furthermore, with the strongest support by evidence-based medicine among the current limited oral treatments of CM, topiramate was administrated every day for 11 days from 2 days before the first NTG injection. The results showed that topiramate partially improved the photophobia behavior of CM models in the short-term with gradually weakened efficacy as the course of the disease prolonged. Meanwhile, NTG-induced increase in Egr1 expression was completely reversed in TNC, SSp, and VISp and partially reduced in LA and TEa by topiramate at the same time point mentioned above. In conclusion, the current results suggested that the abnormal hyperactivities in TNC, SSp and VISp were associated with the pathogenesis of CM.

## Introduction

Migraine is considered one of the most common types of primary headache disorders. Based on the number of monthly headache days (MHDs), it can be classified as episodic migraine (EM) or chronic migraine (CM). CM is defined by the International Classification of Headache Disorders (third edition, ICHD-3) as headache occurring on ≥15 days per month for more than 3 months, with ≥8 days per month meeting criteria for migraine (Headache Classification Committee of the International Headache Society (IHS), 2018). The global prevalence of CM is approximately 2%, among which 75% evolved from EM. Dismally, owning to the poor response to drugs, limited treatment modalities, and diversity of concomitant symptoms, CM has greater impact on personal, social and socioeconomic wellbeing than EM (Buse et al., 2012).

The specific mechanisms of migraine chronification have not been clear, although multiple factors have been suggested, such as central sensitization, peripheral sensitization, abnormal cortical excitability, and abnormal network of pain modulation (May and Schulte, 2016). With the development of imaging technology in recent years, there has been an increasing number of related studies on CM. Summary of clinical imaging researches demonstrates that the functional and structural changes of the brainstem, hypothalamus, thalamus, basal ganglia, and cortex are related to the occurrence of pain (Chen et al., 2020). However, there were differences in the results of the same brain area in different studies (Coppola et al., 2017; Neeb et al., 2017; Planchuelo-Gomez et al., 2020), which may due to the diversity in patients’ background or image processing methods.

Early growth response gene 1 (Egr1), also named zif268 (zinc finger binding protein clone 268), belongs to the category of immediate early genes (IEG). It codes a transcription factor protein, expression of which is induced by neural activity. Egr1 was originally identified in cell cultures, and then it was demonstrated to be activated by various stimuli and its expression was considered as a reporter of neuronal activity. Egr1-enhanced green fluorescent protein (EGFP) transgenic mice were used to apply the endogenous fluorescent signal to indicate neural activity in the brain at cellular resolution (Xie et al., 2014). Previous study has demonstrated that cortical spreading depression (CSD), which might be involved in the biological process of migraine attack, up-regulated Egr1 expression in the somatosensory cortex (Sosthenes et al., 2019). However, there is no research on the expression of Egr1 in the whole brain of CM model.

To generate a detailed abnormal neuronal activity map in the whole brain of CM at cellular resolution, we established CM model using Egr1-EGFP transgenic mice, in which EGFP expression is controlled by Egr1 gene promoter. This study aimed to construct a mouse model of CM with stable modeling markers, as well as to identify brain regions involved in CM. Meanwhile, we hoped to provide a reference for possible therapeutic targets in the future by exploring the brain activity that might be related to different symptoms.

## Materials and Methods

### Animals

A total of 83 mice were used in this study. All animal experiments were carried out in accordance with the guidelines approved by Shanghai Jiao Tong University School of Medicine Institutional Animal Care and Use Committee. BAC-Egr1-EGFP [Tg (Egr1-EGFP) GO90Gsat/Mmucd, from Gensat project, distributed from Jackson Laboratories] were group-housed (3–4 per cage) under standard laboratory conditions [20–22°C, 65–70% relative humidity and a 12 h light/dark cycle (07:00 am–07: 00 pm)], with food and water available ad libitum. Female mice (8–10 weeks) were included in the experiment based on previous research (Pradhan et al., 2014). Animals were randomly assigned to different treatment groups. Weight was recorded at the time before drug injection on each test day for all the experiments.

### Drug Administration

**FIGURE 1:**
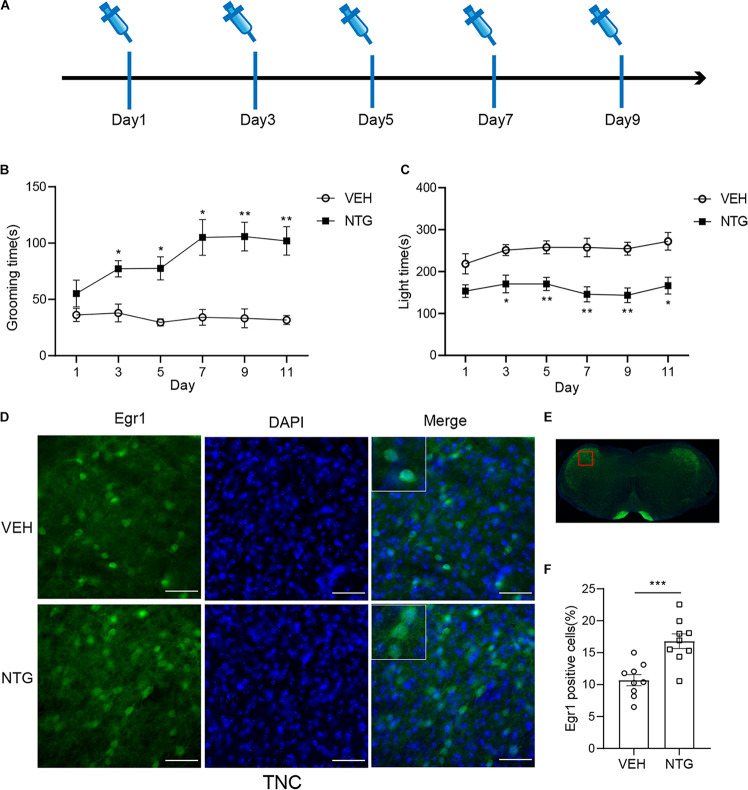
Repeated NTG injection induced migraine related behaviors and elevation of Egr1 expression in TNC **(A)** Drug (NTG/vehicle) administration schedule. **(B)** Head grooming time increased in NTG-injected mice as compared to the control group. **(C)** Chronic injection of NTG decreased time in light chamber in light aversion assay. Two-way ANOVA followed by post-hoc Sidak’s multiple comparison test. **(D,F)** TNC showed significant increase of the number of Egr1 positive cells after CM. **(E)** Schematic representation of the analyzed area. Student’s *t*-test. *n* = 8/group for behavior test and *n* = 3/group for the immunofluorescence. *p < 0.05, **p < 0.01, ***p < 0.001 compared with vehicle. Scale bars = 50 μm. Egr1, early growth response protein 1; TNC, trigeminal nucleus caudalis.

Nitroglycerin (NTG) was bought from Beijing Reagent with a stock solution of 5 mg/ml. Fresh diluted NTG with saline (1 mg/ml) was administered intraperitoneally (i.p.) at a dose of 10 mg/kg every other day for 9 days (i.e., days 1, 3, 5, 7, and 9, Figure 1A). An equivalent volume of saline was used as the vehicle. For the topiramate experiment, mice were i.p. injected with topiramate diluted with saline (1 mg/ml) at a dose of 30 mg/kg or vehicle every day for 11 days (from 2 days before the first NTG injection, Figure 5A), referring to the published data (Pradhan et al., 2014; Greco et al., 2018). On days 3,5,7,9, and 11 of this treatment, topiramate was injected 1h 15 min following NTG injection, and post-drug behavior responses were recorded 45 min later (2 h post NTG).

**FIGURE 2:**
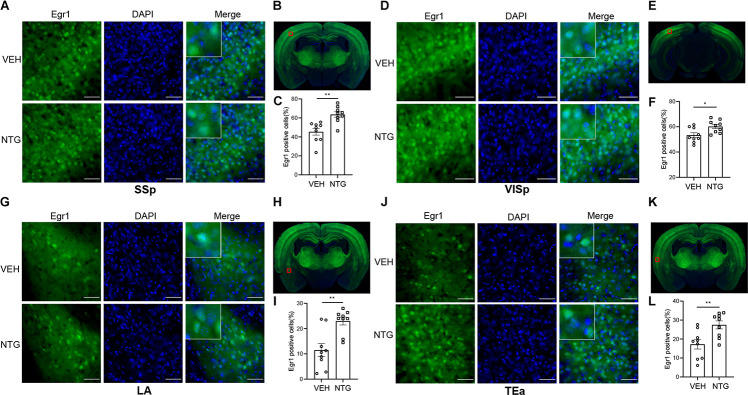
Egr1 expression in SSp, VISp, LA and TEa. More Egr1 positive cells were found in SSp **(A,C)**, VISp **(D,F)**. LA **(G,I)** and TEa **(J,L)** of CM models compared with the control group. **(B,E,H,K)** Schematic representations of the analyzed areas. Student’s *t*-test. *n* = 3/group. *p < 0.05, **p < 0.01 compared with vehicle. Scale bars = 50 μm. Egr1, early growth response protein 1; SSp, primary somatosensory cortex; VISp, primary visual area; LA, lateral amygdala nucleus; TEa, temporal association areas.

**FIGURE 3:**
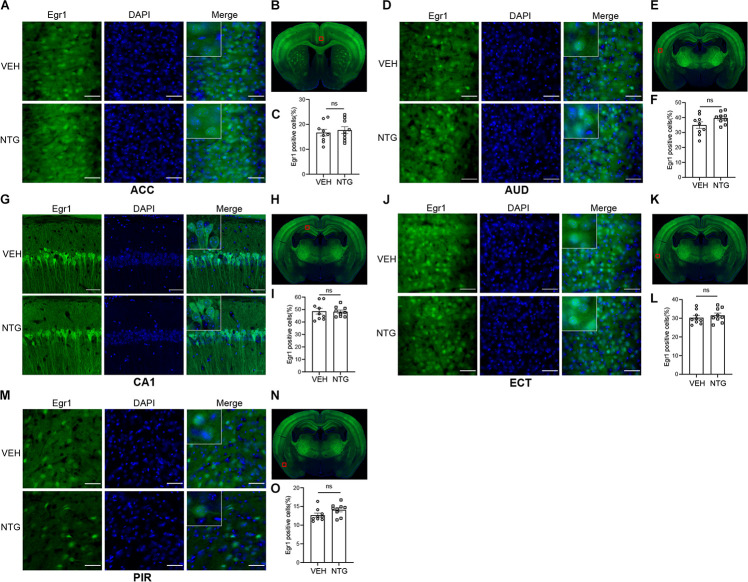
Egr1 expression in ACC, AUD, CA1, ECT and PIR. No changes of Egr1 reactivity were observed in ACC **(A,C)**, AUD **(D,F)**, CA1 **(G,I)**, ECT **(J,L)** and PIR **(M,O)** of CM group compared with vehicle group. **(B,E,H,K,N)** Schematic representations of the analyzed areas. Student’s *t*-test. *n* = 3/group. Scale bars = 50 μm. Egr1, early growth response protein 1; ACC, anterior cingulate area; AUD, auditory areas; ECT, ectorhinal area; PIR, piriform.

**FIGURE 4:**
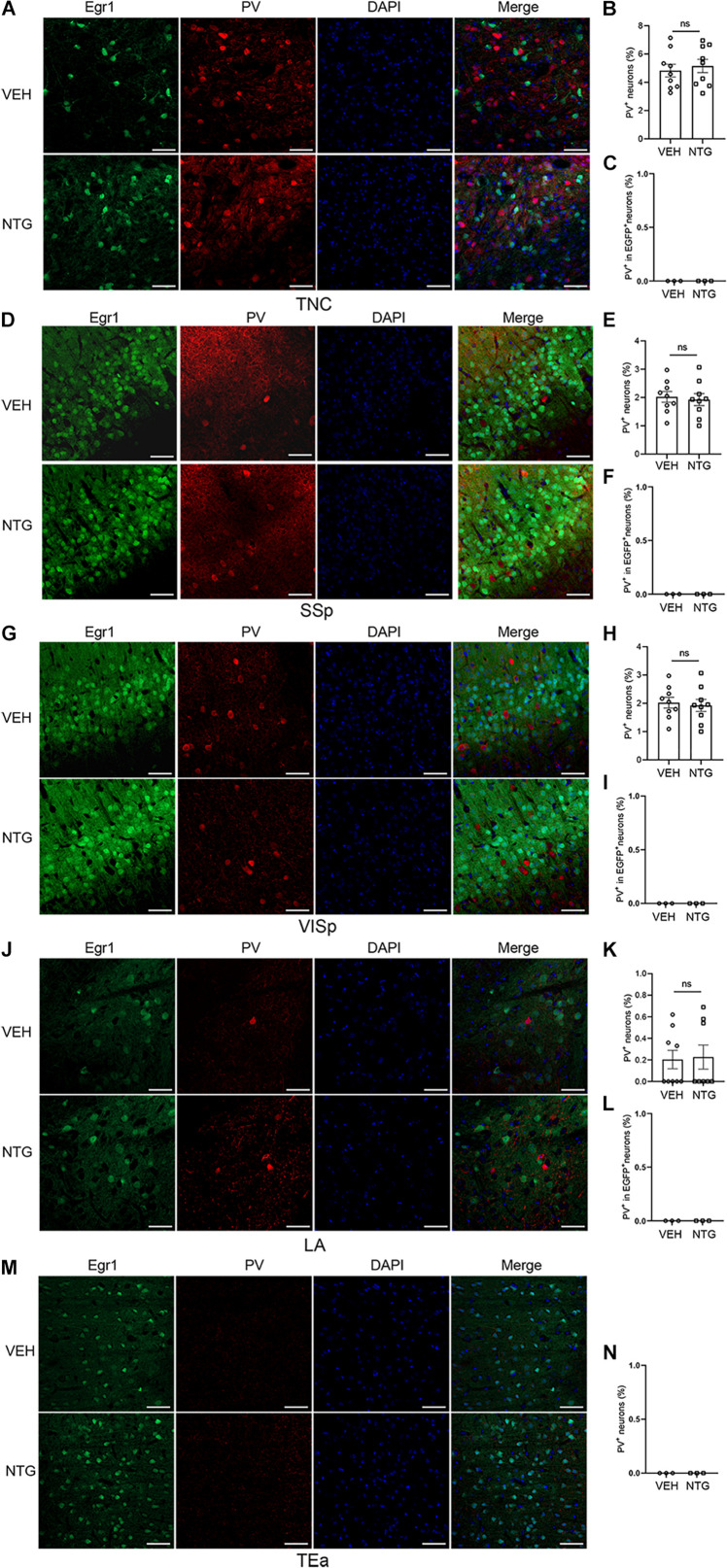
Most of the Egr1-EGFP positive neurons in neocortex and LA were not PV^+^ interneurons. Immunofluorescence staining images were shown on the left **(A,D,G,J,M)**. Quantitation of PV^+^ neurons **(B,E,H,K,N)** and the data of PV^+^ - EGR1^+^ co-localization **(C,F,I,L)** are shown on the right. Student’s *t*-test. *n* = 3/group. Scale bars = 50 μm. Egr1, early growth response protein 1; TNC, trigeminal nucleus caudalis; SSp, primary somatosensory cortex; VISp, primary visual area; LA, lateral amygdala nucleus; TEa, temporal association areas.

**FIGURE 5:**
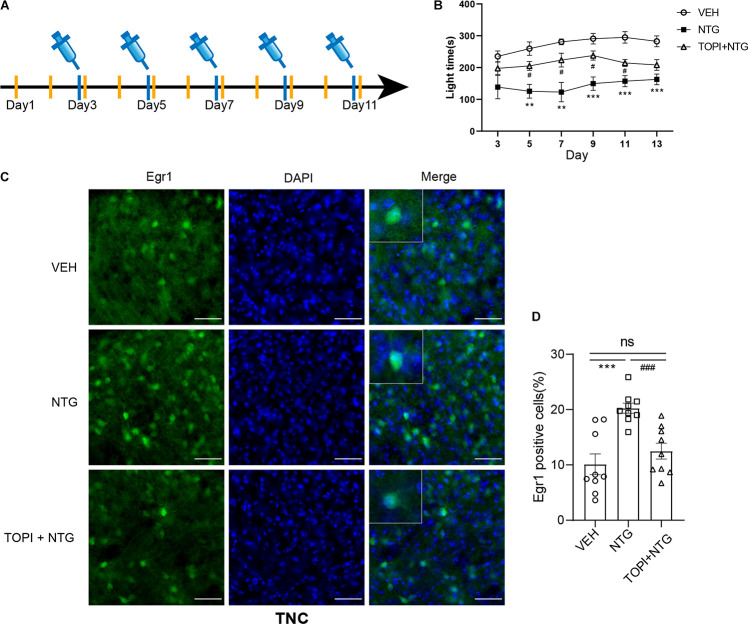
Topiramate improved the photophobia behavior of CM mice and reversed the up-regulation of Egr1 in TNC induced by NTG. **(A)** Drug administration schedule in topiramate experiment. The yellow vertical line represents the time points of topiramate injection. **(B)** Topiramate could partially improve the photophobia behavior of CM mice in the short-term. Two-way ANOVA followed by post-hoc Sidak’s multiple comparison test. **(C,D)** The increased number of Egr1 positive cells in TNC after CM could be reversed by topiramate. Student’s *t*-test. *n* = 8/group for behavior test and *n* = 3/group for the immunofluorescence. **p < 0.01, ***p < 0.001 compared with vehicle. ^#^*p* < 0.05, ^###^*p* < 0.001 compared with NTG. Scale bars = 50 μm. Egr1, early growth response protein 1; TNC, trigeminal nucleus caudalis.

### Behavioral Tests

Eight mice were included in each group. All the tests were operated with multi conditioning system (TSE Systems GmbH, Germany). Mice were extensively handled by the experimenters for 1 week and were well-habituated to the test apparatus for 30 min on the day prior to the experiment and before each test. Behavioral tests were carried out between 12:00 am and 08:00 pm in a quiet room by a blinded experimenter to the treatments. Two or three mice (one from each treatment) were injected each hour, to ensure that each mouse has enough time to be tested at the exact time point.

### Head Grooming Behavior

Animals were placed into a 20 cm × 20 cm transparent box with no cover and allowed to habituate for 30 min, and then their free behavior was video-recorded for 10 min with a camera in front of the box. Head grooming behavior was defined as the bilateral head/face cleaning movement with the forepaws or hind paws. Increased time spent on head grooming was considered as an index of spontaneous pain (Chanda et al., 2013).

### Light Aversion Assay

The light/dark box consisted of two equally-sized chambers, one clear and brightly lit [about 1000 lx] without top, the other one black and fully enclosed which were connected by a small controllable door (8 cm × 6 cm). On test day, mice were individually placed into the middle of the bright chamber for a 1 min acclimation period after which the guillotine-style doors were opened to allow free moving in the entire apparatus for 5 min. Total time spent in the light chamber was recorded. The apparatus was cleaned with 75% ethanol between each test.

### Immunofluorescence

Three mice were included in each group for immunofluorescence. Two hours after the last injection of NTG or vehicle, mice were anaesthetized with 2% pentobarbital sodium and perfused intracardially with phosphate-buffered saline (PBS) and subsequently 4% paraformaldehyde (PFA). The whole brain was removed and post-fixed in 4% PFA at 4°C overnight and transferred to 30% sucrose in PBS for 48h in a refrigerator. Tissues were sectioned using a vibratome at a thickness of 50 μm. Then sections were stained with 4′,6-diamidino-2-phenylindole (DAPI) and mounted on microscope slides. All the slides were placed under a 10× fluoroscope to select three serial slices of the target brain regions for each mouse, referring to the sagittal distance from the Bregma (Supplementary Table 1). Images were acquired by the 20× lens of Olympus VS120 Virtual Microscopy Slide Scanning System. Because intraperitoneal injection was used for systemic administration, the changes in the same area on both sides should be consistent. Thus, we included the left hemisphere for analysis. Areas of 300 μm × 300 μm were extracted in the center of the target brain regions for statistical analysis with GraphPad Prism version 8.0 (Graph Pad Software Inc., San Diego, CA, United States). Numbers of Egr1-positive cells were quantified with QuPath version 0.2.3 (Bankhead et al., 2017) based on the cell size and fluorescence intensity threshold in the regions of interest (ROIs),^1^ with subsequent manual correction.

As for confocal Imaging, sections were incubated in blocking solution (0.1% Triton X100, 1% Bovine Serum Albumin in PBS) with anti-Parvalbumin (PV) rabbit polyclonal antibody (Abcam, 1: 500) for 12 h at 4°C. After series of PBS washes, sections were stained using Alexa Fluor 555 goat anti-rabbit secondary antibody (Invitrogen, 1:1,000 dilution) and DAPI (Beyotime, 1:10,000 dilution) diluted in PBS for 45 min at 37°C. Sections were mounted on slides after washing. Digital images were obtained using a Zeiss (LSM800) microscope with 20× air immersion objectives. The number of PV and Egr1-EGFP double positive neurons was counted manually. All counting was performed by a blinded experimenter who was unaware of the experimental conditions.

### *In vivo* Imaging

In order to identify the character of Egr1^+^ neurons, we crossed our Egr1-EGFP mice with Ai9 mouse line (cre-dependent expression of tdTomato) and PV-cre mouse line or CaMKII-cre mouse line double positive mice to get triple positive mice. Three mice per triple positive type were prepared to receive two-photon intravital imaging of primary visual area (VISp). They received cranial window implantation and were recorded as previously described (Xie et al., 2014).

### Data and Statistical Analysis

The results are presented as Means ± standard error of the mean (SEM). Behavioral results were compared using a two-way ANOVA followed by *post hoc* Sidak’s multiple comparison test, with drug and time as factors. Differences in numbers of Egr1 positive cells between two groups were determined by Student’s *t*-test. Actual *p* values were given and statistically significant was considered at *p* < 0.05 in all cases.

## Results

### Repeated NTG Injection Induced Photophobia and Increased Head Grooming Time

Consistent with previous studies (Zhang et al., 2020), we observed that in mice treated with NTG, head grooming time increased as compared with the vehicle control group on days 3, 5, 7, and 9. Moreover, significant change was detected on day 11 (2 days after stopping NTG injection) (Figure 1B and Supplementary Table 2). Head grooming time significantly increased in NTG treated group on day 3, 5, 7, and 9. And this abnormal grooming time could even be detected in 2 days later after the last NTG injection. Chronic injection of NTG also decreased the time in light chamber on days 3, 5, 7, and 9 as compared with vehicle control group and the change existed 2 days after the last NTG injection (Figure 1C and Supplementary Table 3).

### Elevation of Egr1 in Brain Regions After CM Induction

In order to detect the neural activity in brain regions that might be associated with CM, we examined the expression of Egr1 in multiple brain regions. After CM induction, the number of Egr1 positive cells significantly increased in TNC (*p* = 0.006, VEH: 10.697 ± 0.872 vs. NTG: 16.798 ± 1.149, Figures 1D–F), which is a primary structure for receiving noxious stimuli. What’s more noteworthy was that, in addition to the changes in the brain regions that were clearly related to migraine, more Egr1 positive cells were found in primary somatosensorycortex (SSp) (*p* = 0.001, VEH: 45.369 ± 3.544 vs. NTG: 63.640 ± 2.858, Figures 2A–C), VISp (*p* = 0.0153, VEH: 53.339 ± 1.919 vs. NTG: 60.192 ± 1.642, Figures 2D–F), lateral amygdala nucleus (LA) (*p* = 0.0015, VEH: 11.498 ± 2.569 vs. NTG: 22.991 ± 1.558, Figures 2G–I), and temporal association areas (TEa) (*p* = 0.0063, VEH: 17.237 ± 2.485 vs. NTG: 27.482 ± 2.116, Figures 2J–L) compared with the control group. No changes of Egr1 expression were observed in anterior cingulate area (ACC) (*p* = 0.6057, VEH: 16.680 ± 1.344 vs. NTG: 17.692 ± 1.373, Figures 3A–C), auditory areas (AUD) (*p* = 0.0831, VEH: 34.990 ± 2.190 vs. NTG: 39.685 ± 1.287, Figures 3D–F), CA1 (*p* = 0.8803, VEH: 48.686 ± 2.306 vs. NTG: 48.279 ± 1.334, Figures 3G–I), ectorhinal area (ECT) (*p* = 0.4774, VEH: 30.287 ± 1.188 vs. NTG: 31.541 ± 1.249, Figures 3J–L) and piriform (PIR) (*p* = 0.0816, VEH: 12.662 ± 0.555 vs. NTG: 14.115 ± 0.551, Figures 3M–O).

### Egr1-EGFP^+^ Neurons Were Mostly Excitatory

Quantification of the percentage of PV^+^ or CaMKII^+^ neurons in Egr1-EGFP^+^ neurons was conducted after in vivo imaging. The result showed that most Egr1-EGFP^+^ neurons are also CaMKII^+^ (97.85%, 58341 of 59620 neurons), and only 0.03% (38 of 125,846 neurons) are PV^+^ (Supplementary Figure 1). Furthermore, EGFP-positive cells in the brain regions responsing to NTG had rarely co-localization with PV (Figures 4C,F,I,L,N), which had no significant change after CM induction, indicating interneurons were unlikely to be involved in this process or had negligible effect (TNC: *p* = 0.6255, VEH: 4.827 ± 0.450 vs. NTG: 5.150 ± 0.470, Figures 4A,B; SSp: *p* = 0.7359, VEH: 2.022 ± 0.190 vs. NTG: 1.924 ± 0.213, Figures 4D,E; VISp: *p* = 0.5731, VEH: 4.677 ± 0.202 vs. NTG: 4.509 ± 0.210, Figures 4G,H; LA: *p* = 0.8706, VEH: 0.203 ± 0.085 vs. NTG: 0.226 ± 0.111, Figures 4J,K; TEa: VEH:0 ± 0 vs. NTG: 0 ± 0, Figure 4M).

### Topiramate Partially Improved the Photophobia Behavior of CM Mice

In light of the fact that NTG-induced photophobia and the light aversion assay showed a more significant difference than head-grooming behavior, we examined the effect of topiramate, a first-line medicine to treat CM, on photophobia in CM model mice. While topiramate partially improved the NTG-induced photophobia behavior on days 3, 5, 7, 9, and 11 (Figure 5B and Supplementary Table 4), photophobia time showed no difference between the CM group and the topiramate treatment group on day 13. These data suggested that topiramate had the effect of partially improving the photophobia behavior of mice at the initial stage of onset, but its efficacy gradually weakened as the course of disease prolonged.

### NTG-Induced Increase in Egr1 Expression Could Be Reversed by Topiramate

**FIGURE 6:**
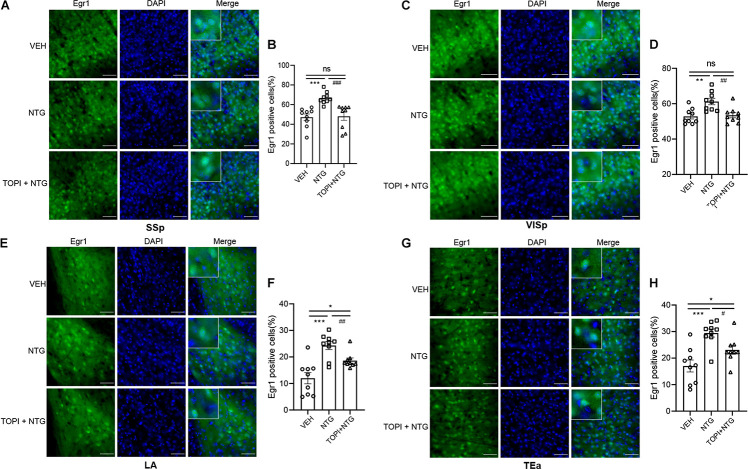
Egr1 expression in SSp, VISp, LA, and TEa of CM model after topiramate treatment. The increased Egr1 expression induced by NTG was decreased to varying degrees by topiramate in these regions. **(A–F)** There was no statistical difference between the control group and topiramate + NTG group in the SSp and VISp. **(G,H)** The numbers of Egr1 positive cells in the topiramate + NTG group were still higher in LA and TEa than that in the control group. Student’s t test. n = 3/group. *p < 0.05, **p < 0.01 compared with vehicle. ^#^*p* < 0.05, ^##^*p* < 0.01, ^###^*p* < 0.001 compared with NTG. Scale bars = 50 μm. Egr1, early growth response protein 1; SSp, primary somatosensory cortex; VISp, primary visual area; LA, lateral amygdala nucleus; TEa, temporal association areas.

We then tested the effect of topiramate on Egr1 expression in CM mice. The result showed that not only in TNC (VEH vs. NTG: *p* = 0.0002, NTG vs. TOPI + NTG: *p* = 0.0003, VEH: 10.112 ± 1.882, NTG: 20.247 ± 0.917, TOPI + NTG: 12.487 ± 1.433, Figures 5C,D) but also in SSp (VEH vs. NTG: *p* = 0.0001, NTG vs. TOPI + NTG: *p* = 0.0009, VEH: 47.205 ± 3.275, NTG: 66.779 ± 1.992, TOPI + NTG: 48.130 ± 4.121, Figures 6A,B), VISp (VEH vs. NTG: *p* = 0.0022, NTG vs. TOPI + NTG: *p* = 0.0042, VEH: 52.806 ± 1.440, NTG: 61.248 ± 1.809, TOPI + NTG: 53.474 ± 1.473, Figures 6C,D), LA (VEH vs. NTG: *p* = 0.0002, NTG vs. TOPI + NTG: *p* = 0.0067, VEH: 11.994 ± 2.104, NTG: 24.304 ± 1.538, TOPI + NTG: 18.624 ± 0.979, Figures 6E,F), and TEa (VEH vs. NTG: *p* = 0.0003, NTG vs. TOPI + NTG: *p* = 0.0115, VEH: 17.027 ± 2.251, NTG: 29.460 ± 1.528, TOPI + NTG: 23.091 ± 1.629, Figures 6G,H), the increased Egr1 expression induced by NTG also decreased to varying degrees. It should be noted that no difference was found in TNC (*p* = 0.3305), SSp (*p* = 0.8628), and VISp (*p* = 0.7500) between the control group and topiramate + NTG group. Interestingly, the numbers of Egr1 positive cells were still higher in LA (*p* = 0.0114) and TEa (*p* = 0.0443) in the topiramate + NTG group than those in the control group, indicating that the up-regulation of neural activity in these two brain regions could not be completely reversed by topiramate.

## Discussion

It is believed that migraine is a primary headache caused by the combined action of CSD and trigeminal neurovascular dysfunction (Brennan and Pietrobon, 2018). After the occurrence of CSD, noxious stimuli from the meninges, blood vessels, and trigeminal nerve innervation are transmitted through the trigeminal ganglion to the trigeminocervical complex, then ascend to the brainstem, thalamus, hypothalamus, and basal ganglia, and finally project to multiple cortical areas, which are involved in the processing of noxious information and cause headaches and related symptoms (Akerman et al., 2011). Although numerous researches studied the nerve conduction pathways that might be involved in the occurrence of migraine, the results were not consistent.

Repeated intraperitoneal injection of NTG is currently a common method for establishing CM mice models (Pradhan et al., 2014). Consistent with previous studies (Farajdokht et al., 2017; Zhang et al., 2020), the animal model using Egr1-EGFP transgenic mice in this study well replicated the behavioral characteristics of CM. Considering Egr1-EGFP^+^ neurons were mostly excitatory here, we speculated that this was an appropriate animal model for studying neuronal activity. As an important structure involved in the pathogenesis of migraine, TNC showed increased Egr1 expression in this CM mouse model (Noseda and Burstein, 2013). In addition, we observed increased neuronal activity in brain regions in SSp, VISp, LA, and TEa. Topiramate, with the strongest support by evidence-based medicine among the current limited oral treatments of CM (Dodick, 2018), inhibited those changes to varying degrees.

Photophobia is a common accompanying symptom in migraine patients. Previous electrophysiological studies demonstrated that after the activation of retinal photoreceptors caused by light stimulation, the photic signals were transmitted to thalamus via trigeminovascular neurons, and then the axons of the neurons projected to multiple areas including somatosensory and visual cortices (Noseda et al., 2019). Consistent with these, we observed increased neuronal activity in VISp and SSp in our study. This enhancement may be related to inherent defect in cortical habituation or in ionic channels that modulate excitability (Bernstein et al., 2019). Many studies have shown the effectiveness of topiramate on the photophobia symptoms of migraine (Dodick et al., 2009; Silberstein et al., 2009). However, only a transient and partial reduction of behaviors are observed in our research, which led us to consider if other factors were involved in the persistence of photophobia.

Migraine with psychiatric comorbidity implies heavier personal and societal burden. More than half of migraineurs meet at least one diagnostic criteria for anxiety disorder, and the incidence of anxiety is about twice that of depression. Mental disorders are more common in CM patients than in EM patients (Minen et al., 2016). However, mechanisms underlying the etiology of mental disorders in migraine patients remain to be clarified. Previous study has suggested the value of LA in the processes of noxious stimuli (Blair et al., 2005). Moreover, a significantly smaller volume was detected by MRI in LA of the patients with mental disease (Asami et al., 2018). In this study, neuronal activity increased in LA and whether the brain regions that receive its signal input also respond accordingly requires further research. More fancifully, neural activity in TEa, which is associated with mental imagery, also increased. Mental imagery is a process that integrates multisensory perception to generate a vivid mental image or experience (Di Corrado et al., 2019). TEa was found to have an increased functional connection with insula in a migraine rat model recently, which also suggested the important role of it (Jia et al., 2020). In our study, the enhanced neuronal activity in LA and TEa could not be completely reversed by topiramate. In the light/dark box test, which underlies anxiety-like behavior, photophobia was also partially relieved in the initial stage. These results indicate that the long-term treatment effect of topiramate is sub-optimal, and it may be reasonable to add antidepressants to treat related symptoms, which is also in line with clinical treatment experience (Amoozegar, 2017).

It is thought that the pharmacologic mechanisms of the antimigraine activity of topiramate may include regulation of cell membrane ion channels in cell membrane (voltage-gated sodium, calcium channel, and potassium channel) and modulation of neurotransmitter release (glutamate and c-aminobutyric acid) (Aurora and Brin, 2017). Based on these mechanisms of action, topiramate may prevent the development of CSD by reducing nociceptive transmission and inhibiting neuronal hyperexcitability. On the other hand, although the exact biological mechanism by which migraineurs are susceptible to depression is not clear. There is evidence suggesting several potential pathways may be involved. These include serotonergic dysfunction, reduced tyramine conjugation, ovarian hormone variation, and hypothalamic-pituitary adrenal axis dysregulation (Antonaci et al., 2011). It can be seen that topiramate mainly modulates the neurotransmitter that related to CSD, and the transmitter changes in CM combined with mental disorders may exist until new modulating drugs are added.

According to previous studies, migraineurs had concordant decreases in the gray matter volume of ACC and activation of it was also found (Jia and Yu, 2017). However, our results did not show changes of neuronal activity in ACC, probably because CM can incorporate one or more different accompanying symptoms that might be related to ACC with varying degrees, such as cognitive disorder, nociception, and dysphrenia, which may be the results of different activity status of ACC (Jia et al., 2019). Apart from photophobia, migraine is associated with derangements in perception of other sensory modalities including hearing and smell, so we measured the neuronal activity in AUD, PIR, and ECT, but the results were negative. Combining previous studies with different conclusions (Harriott and Schwedt, 2014), we must realize that the processing of sensory information of CM is a complicated process that requires in-depth research. Besides, considering that migraine may be a risk factor for transient global amnesia (TGA) which had lesions located in hippocampal CA1 region (He et al., 2021), CA1 was also analyzed in this study. The results suggested that CA1 probably didn’t participate in the pathological process of CM.

In our study, a CM model was stably established by interval and repeated NTG injection in Egr1-EGFP transgenic mice, and brain activity changes that might be related to different concomitant symptoms were found at the cellular level. This allows further identification of potential drug targets to alleviate CM at cellular specificity. However, our study still has some limitations. First, changes in neuronal activity could only be observed in space but not in time, so that the neural projection pathways could not be concluded; Second, the low expression of Egr1 in hypothalamus limited the research on this region; Third, the possible differences in neuronal activity between sexes could not be detected. which might be related to the difference in prevalence.

Nonetheless, this model indicated changes of neuronal activity in the specific brain regions involved in CM, identifying the specific function of these neurons might benefit for studying the pathology of CM. Moreover, manipulating these neurons might be potential target for the treatment of CM. Taken together, this study screened and provided neuronal activity data of target brain regions in a migraine mouse model.

## Data Availability Statement

The original contributions presented in the study are included in the article/Supplementary Material, further inquiries can be directed to the corresponding author.

## Ethics Statement

The animal study was reviewed and approved by Shanghai Jiao Tong University School of Medicine Institutional Animal Care and Use Committee.

## Author Contributions

YW and J-SG contributed to study design and final revision of the manuscript. FW was in charge of experiment performance and manuscript drafting. WJ and LG collected and analyzed the experimental data. CL and MD revised the manuscript. XR and CZ provided the interpretation of experimental data. All authors contributed to the article and approved the submitted version.

## Conflict of Interest

The authors declare that the research was conducted in the absence of any commercial or financial relationships that could be construed as a potential conflict of interest.

## Publisher’s Note

All claims expressed in this article are solely those of the authors and do not necessarily represent those of their affiliated organizations, or those of the publisher, the editors and the reviewers. Any product that may be evaluated in this article, or claim that may be made by its manufacturer, is not guaranteed or endorsed by the publisher.

## Footnotes

1
https://qupath.github.io
