# Supplementary material for: Detecting Abnormal Neuronal Activity in a Chronic Migraine Model by Egr1-EGFP Transgenic Mice
Source: Front Neurosci. 2021 Aug 12;15:705938. doi: 10.3389/fnins.2021.705938 (PMC8387874; doi:10.3389/fnins.2021.705938)
Supplement: Supplementary file 1 [file Table_1.DOCX]

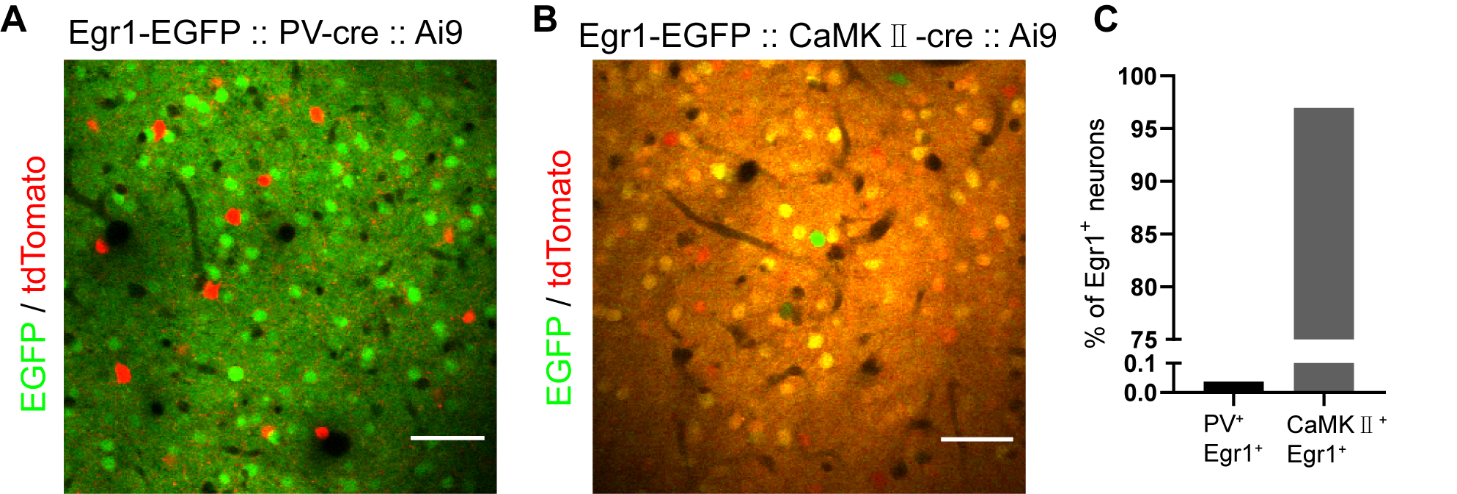


**Supplementary FIGURE 1 |** Quantification of the percentage of PV^+^ or CaMKII^+^ neurons in EGR1-EGFP^+^ neurons. The result showed that most Egr1-EGFP+ neurons are also CaMKII^+^ (97.85%, 58341 of 59620 neurons), and only 0.03% (38 of 125846 neurons) are PV^+^.

| Brain regions | Sagittal distance from Bregma (mm) | Brain regions | Sagittal distance from Bregma (mm) |
| --- | --- | --- | --- |
| TNC | -7.32 | SSp（Ⅳ） | -1.70 |
| VISp（Ⅳ） | -3.80 | LA | -1.70 |
| TEa（Ⅳ） | -2.06 | ACC | 1.18 |
| AUD（Ⅳ） | -2.06 | CA1 | -1.82 |
| ECT（Ⅴ） | -1.70 | PIR | -1.82 |

**Supplementary TABLE 1** **|** The sagittal distance of the target brain regions from Bregma. Egr1, early growth response protein 1; TNC, trigeminal nucleus caudalis; SSp, primary somatosensory cortex; VISp, primary visual area; LA, lateral amygdala nucleus; TEa, temporal association areas; ACC, anterior cingulate area; AUD, auditory areas; ECT, ectorhinal area; PIR, piriform.

| Day | VEH  (Mean±SEM) | NTG  (Mean±SEM) | *P* value  (VEH vs. NTG) |
| --- | --- | --- | --- |
| 1 | 36.188±5.845 | 55.125±11.963 | 0.7068 |
| 3 | 37.825±7.920 | 77.125±7.257 | 0.0157 |
| 5 | 29.563±3.078 | 77.500±10.292 | 0.0117 |
| 7 | 34.000±7.101 | 105±15.913 | 0.0142 |
| 9 | 33.125±8.459 | 105.750±12.839 | 0.0029 |
| 11 | 31.625±4.097 | 101.875±12.719 | 0.0039 |

**Supplementary TABLE 2** **|** Numerical value and statistics of Figure 1B.

| Day | VEH  (Mean±SEM) | NTG  (Mean±SEM) | *P* value  (VEH vs. NTG) |
| --- | --- | --- | --- |
| 1 | 218.375±24.068 | 153.500±15.052 | 0.2256 |
| 3 | 251.125±13.031 | 170.500±21.060 | 0.0420 |
| 5 | 257.750±15.369 | 170.625±15.712 | 0.0085 |
| 7 | 257.500±21.843 | 145.875±17.930 | 0.0093 |
| 9 | 254.500±15.382 | 143.500±17.458 | 0.0019 |
| 11 | 272.000±21.281 | 166.500±19.975 | 0.0169 |

**Supplementary TABLE 3** **|** Numerical value and statistics of Figure 1C.

| Day | VEH  (Mean±SEM) | NTG  (Mean±SEM) | TOPI+NTG  (Mean±SEM) | *P* value  (VEH vs. NTG) | *P* value  (NTG vs. TOPI+NTG) | *P* value  (VEH vs. TOPI+NTG) |
| --- | --- | --- | --- | --- | --- | --- |
| 3 | 235.250±17.051 | 138.625±36.955 | 197.625±18.728 | 0.0913 | 0.3711 | 0.3474 |
| 5 | 259.375±21.021 | 125.500±21.670 | 205.125±13.786 | 0.0015 | 0.0234 | 0.1193 |
| 7 | 280.750±9.227 | 122.750±30.390 | 223.375±21.801 | 0.0025 | 0.0464 | 0.0864 |
| 9 | 290.875±16.726 | 149.625±21.222 | 214.625±11.071 | 0.0004 | 0.0122 | 0.0749 |
| 11 | 294.750±18.217 | 157.000±17.479 | 208.250±16.591 | 0.0002 | 0.0410 | 0.0075 |
| 13 | 282.375±17.196 | 162.750±16.587 | 208.250±16.591 | 0.0005 | 0.1644 | 0.0200 |

**Supplementary TABLE 4** **|** Numerical value and statistics of Figure 5B.
